# Macular Cone Abnormalities in Retinitis Pigmentosa with Preserved Central Vision Using Adaptive Optics Scanning Laser Ophthalmoscopy

**DOI:** 10.1371/journal.pone.0079447

**Published:** 2013-11-19

**Authors:** Yukiko Makiyama, Sotaro Ooto, Masanori Hangai, Kohei Takayama, Akihito Uji, Akio Oishi, Ken Ogino, Satoko Nakagawa, Nagahisa Yoshimura

**Affiliations:** Department of Ophthalmology and Visual Sciences, Kyoto University Graduate School of Medicine, Kyoto, Kyoto, Japan; University Hospital La Paz, Spain

## Abstract

**Purpose:**

To assess macular photoreceptor abnormalities in eyes with retinitis pigmentosa (RP) with preserved central vision using adaptive optics scanning laser ophthalmoscopy (AO-SLO).

**Methods:**

Fourteen eyes of 14 patients with RP (best-corrected visual acuity 20/20 or better) and 12 eyes of 12 volunteers underwent a full ophthalmologic examination, fundus autofluorescence, spectral-domain optical coherence tomography (SD-OCT), and imaging with a prototype AO-SLO system. Cone density and spatial organization of the cone mosaic were assessed using AO-SLO images.

**Results:**

In 3 eyes with RP and preserved central vision, cones formed a mostly regular mosaic pattern with small patchy dark areas, and in 10 eyes, the cone mosaic patterns were less regular, and large dark regions with missing cones were apparent. Only one eye with RP demonstrated a normal, regular cone mosaic pattern. In eyes with RP, cone density was significantly lower at 0.5 mm and 1.0 mm from the center of the fovea compared to normal eyes (*P*<0.001 and 0.021, respectively). At 0.5 mm and 1.0 mm from the center of the fovea, a decreased number of cones had 6 neighbors in eyes with RP (*P* = 0.002 for both). Greater decrease in cone density was related to disruption of the photoreceptor inner segment (IS) ellipsoid band on SD-OCT images (*P* = 0.044); however, dark regions were seen on AO-SLO even in areas of continuous IS ellipsoid on SD-OCT. Decreased cone density correlated thinner outer nuclear layer (*P* = 0.029) and thinner inner segment and outer segment thickness (*P* = 0.011) on SD-OCT.

**Conclusions:**

Cone density is decreased and the regularity of the cone mosaic spatial arrangement is disrupted in eyes with RP, even when visual acuity and foveal sensitivity are good. AO-SLO imaging is a sensitive quantitative tool for detecting photoreceptor abnormalities in eyes with RP.

## Introduction

Retinitis pigmentosa (RP), the prevalence of which has been reported as approximately 1∶4,000 worldwide, is the term used for a group of disorders that are characterized by inherited, progressive dysfunction and dystrophy of retinal tissue [1], [2]. Initial involvement of photoreceptors leads to subsequent damage to inner retinal cells. The age of onset of visual impairment in the different types of RP ranges from infancy to late adulthood. Visual impairment usually manifests as night blindness and visual field loss. The eventual visual burden from retinal dystrophy can range from just sectorial visual field loss to profound loss of the peripheral visual field. Central vision may be well preserved even if electroretinography (ERG) shows remarkably reduced response [3].

Optical coherence tomography (OCT) has become the gold standard for assessing anatomical abnormalities in retinal diseases. Structural changes in the photoreceptors of eyes with RP, such as varying degrees of disruption of inner segment (IS) ellipsoid and thinning of the outer plexiform layer (OPL) and outer nuclear layer (ONL), have been identified using time-domain OCT (TD-OCT) and spectral-domain OCT (SD-OCT) [4]–[10]. These imaging modalities have not, however, provided sufficiently clear images of individual photoreceptor cells to allow identification of a specific structural abnormality that may explain visual disturbance in eyes with RP. The primary reason for this failure is that ocular optics possess aberrations, which can be compensated for by incorporating adaptive optics (AO)—specifically, either OCT or another imaging technique such as scanning laser ophthalmoscopy (SLO)—into the imaging system.

An AO system consists of a wavefront sensor that measures aberrations of the whole eye and a deformable mirror or a spatial light modulator that compensates for these aberrations in living eyes [11]–[15]. The addition of AO to imaging systems such as flood-illuminated ophthalmoscopes or SLO equipment has allowed researchers to obtain clear images of microstructural details in living eyes, including abnormalities in individual cone photoreceptors in patients with various retinal diseases [16]–[31].

Several researchers have reported the observation of abnormal cone patterns using AO-imaging devices in eyes with forms of inherited retinal degeneration [17]–[21]. However, it has not been investigated fully whether macular cone abnormalities occur in RP patients with preserved central vision. In the current study, we used a prototype AO-SLO system to assess macular photoreceptor abnormalities in RP patients with preserved central vision in comparison with findings on SD-OCT images.

## Methods

All investigations adhered to the tenets of the Declaration of Helsinki, and the study was approved by the institutional review board and the ethics committee at Kyoto University Graduate School of Medicine. The nature of the study and its possible consequences were explained to study candidates, after which written informed consent was obtained from all who participated. For a patient under 20 years old (Case 10, 16 y), written informed consent was obtained from both the participant and his father.

### Participants

A total of 26 participants were included in this prospective cross-sectional study. Fourteen were patients (14 eyes, 5 men and 9 women; mean age, 42.6 y; range, 16–63 y) with RP and preserved central vision (best-corrected visual acuity [BCVA] 20/20 or better) but without any other macular abnormality; all patients visited the Kyoto University Hospital, Kyoto, Japan, between July 2011 and March 2012. The other 12 participants were healthy volunteers (12 eyes; 11 men and 1 woman; mean age, 38.2 y; range, 28–52 y) with no eye disease. RP was diagnosed based on the presence of features including night blindness, progressive loss of visual field, fundus appearance (attenuated retinal vessels, mottling and granularity of the retinal pigment epithelium, bone-spicule intraretinal pigmentation, and optic disc pallor), and electroretinographic abnormalities. We excluded eyes with cone-rod or cone dystrophy, Leber congenital amaurosis, retinal inflammatory diseases, autoimmune paraneoplastic retinopathy, or drug toxicity.

### Ophthalmologic Examinations

All subjects underwent, at the same visit for each participant, comprehensive ophthalmologic examination including BCVA assessed with the Landolt chart and expressed as the logarithm of the minimal angle of resolution (logMAR), visual field testing with Humphrey Field Analyzer (HFA) with the 10-2 Swedish Interactive Threshold Algorithm (SITA) standard program, intraocular pressure (IOP); axial length assessed using an IOL Master (Carl Zeiss Meditec, Dublin, CA, USA), indirect ophthalmoscopy, slit-lamp biomicroscopy with a contact lens, color fundus photography, fundus autofluorescence (FAF), SD-OCT, and AO-SLO. FAF images were obtained with confocal scanning (HRA2; Heidelberg Engineering, Dossenheim, Germany) using 30 degrees camera objective. All patients had undergone 30-Hz flicker ERG within the past 2 y. ERG results were recorded according to the ISCEV standard protocol recommended in 2008, using LS-C (Mayo Co., Nagoya, Japan) and Neuropack MEB-2204 systems (Nihon Kohden, Tokyo, Japan) [32]. Notably, one patient had undergone focal macula ERG [33].

### Adaptive Optics Scanning Laser Ophthalmoscopy System

We have developed an original prototype AO-SLO system in collaboration with Canon Inc [34], [35]. The system is composed of 4 primary optical subsystems: the AO subsystem, which includes the wavefront sensor and the spatial light modulator; the high-resolution confocal SLO imaging subsystem; the wide-field imaging subsystem; and the pupil observation subsystem, which facilitates the initial alignment of the subject's pupil with respect to the optical axis of the AO-SLO system through adjustment of the chin rest position. The wavefront sensor measures aberrations in the whole eye, and the spatial light modulator compensates for these aberrations. The details of the AO-SLO system are described in the Information S1. The AO-SLO system is confocal, enabling creation of “en face” images in any plane; these images show individual cone photoreceptor cells.

### Analysis of Adaptive Optics Scanning Laser Ophthalmoscopy Images: Cone Mosaic Features

**Figure 1:**
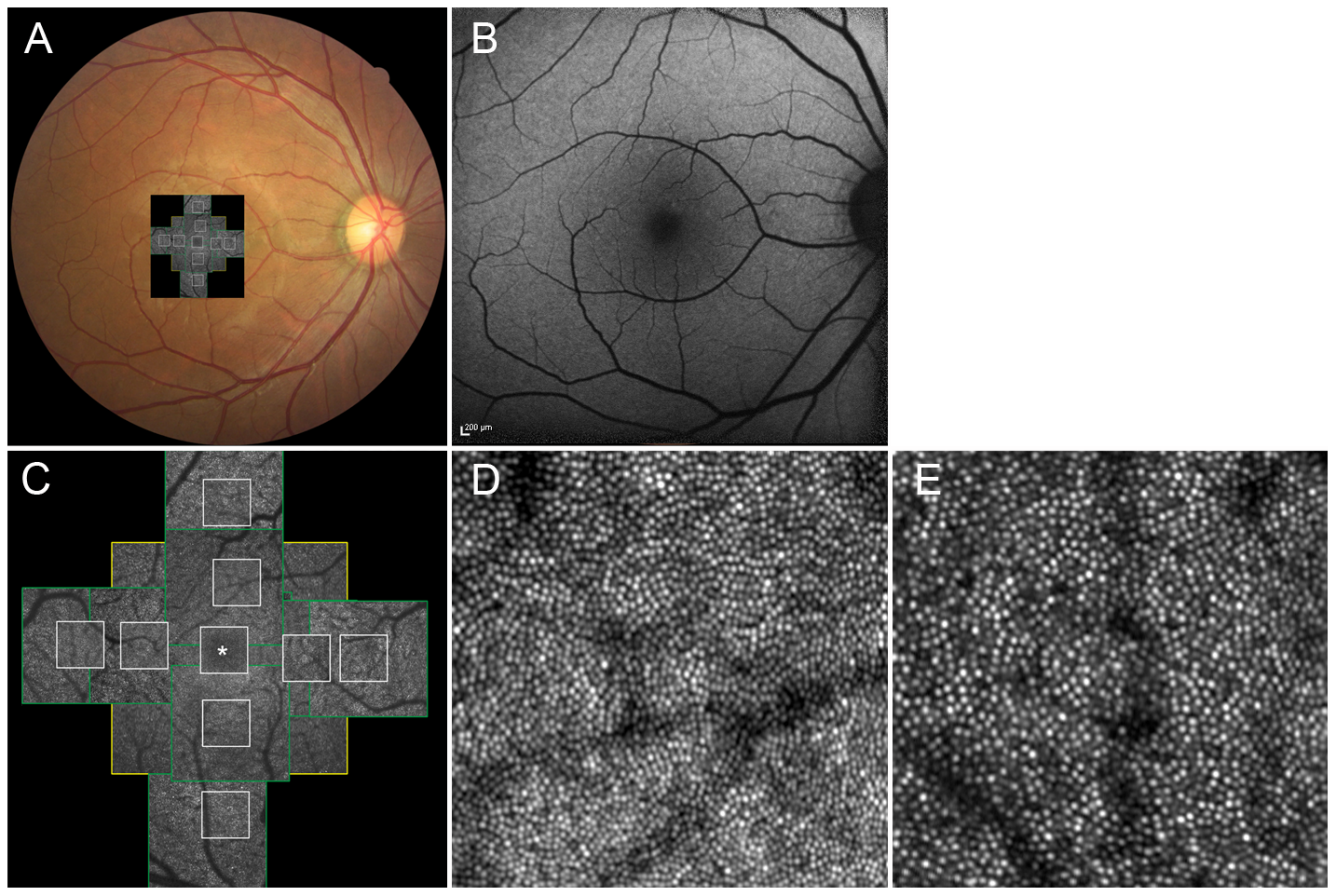
Normal cone photoreceptors. (A) Adaptive optics scanning laser ophthalmoscopy (AO-SLO) image registered on color fundus photograph. (B) Fundus autofluorescence (FAF) imaging. (C) Montage of a series of high-resolution images from the central fovea outward to 1.5 mm from the center of the fovea, obtained by AO-SLO. Asterisk = foveal center. First, 3 different field of view images (L [yellow box]: 1700×1700 µm, M [green box]: 820×820 µm, S [white box]: 340×340 µm) centered on the center of the fovea were obtained, followed by 2 field of view images (M and S) centered 0.5 mm and 1.0 mm from the center of the fovea at each direction (superior, nasal, inferior, temporal) (D and E) Representative images of areas 0.5 mm (D) and 1.0 mm (E) from the center of the fovea (340 µm×340 µm area).

For each eye, we acquired a series of AO-SLO images at each of several locations in the macula. The series at each location was acquired by shifting the focus from the retinal nerve fiber layer (RNFL) to the retinal pigmented epithelium (RPE), with particular attention paid to acquisition of images that showed the cone mosaic. First, 3 different field-of-view images (L: 1700×1700 µm, M: 820×820 µm, S: 340×340 µm) centered on the center of the fovea were obtained, followed by 2 field-of-view images (M and S) centered 0.5 mm and 1.0 mm from the center of the fovea in each direction (superior, nasal, inferior, temporal) (Fig. 1). At each location of interest, 32 images were acquired and averaged to reduce noise. We verified correspondence between each montage and the area of interest by comparing the high-magnification AO-SLO image with the wide-field AO-SLO images for that eye. The montage of AO-SLO images were used for the registration with images of other imaging modalities by matching the shape of the vessels.

To evaluate cones, we applied the automated cone labeling process of Li and Roorda [36]. After automated cone labeling, 2 experienced observers independently examined each image. If cones were visible but had not been labeled, the observer manually labeled the areas where cones were visible and entered this area into the computer software program.

As has been reported for similar systems [11]–[31], we found that our system did not always allow clear visualization of individual cones within much of the central fovea. However, we could clearly distinguish individual cones >0.2 mm from the center of the fovea. Therefore, we obtained an estimate of cone density in areas 0.5 mm and 1.0 mm from the foveal center by instructing the computer software to divide the number of cones in each imaging area by the size of the area. The center of the fovea, defined here as the center of the foveal avascular zone, was determined from the montage of AO-SLO images. We measured cone density in each of 4 directions (superior, lower, nasal, and temporal), and the mean density was calculated from the densities in all 4 directions. To obtain accurate scan lengths, we corrected the magnification effect in each eye using the adjusted axial length method reported by Bennett et al [37].

**Figure 2:**
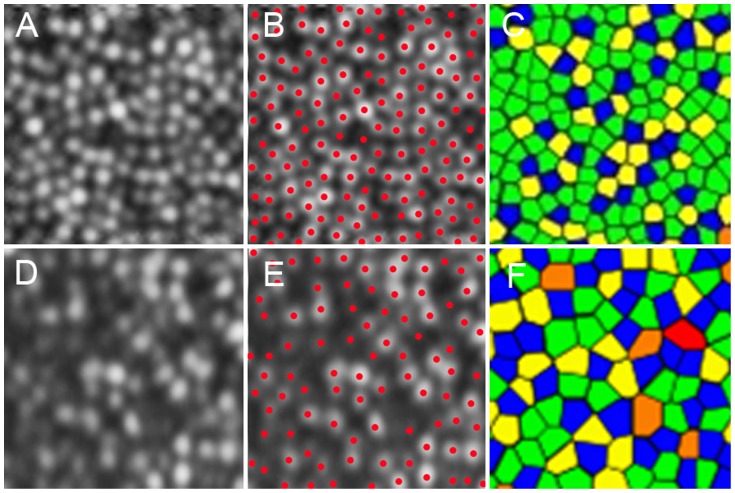
Cone labeling and cone density/arrangement measurement. (A) A cone mosaic image 0.5 mm from the center of the fovea in a normal eye. (B) Cone labeling results. (C) Voronoi diagram. The colors indicate the number of sides of each Voronoi polygon (pink, 4; blue, 5; green, 6; yellow, 7; and orange, 8). (D) A cone mosaic image 0.5 mm from the center of the fovea in an eye with retinitis pigmentosa (RP). (E) Cone labeling results. (F) Voronoi diagram. The colors indicate the number of sides of each Voronoi polygon (pink, 4; blue, 5; green, 6; yellow, 7; and orange, 8). The cone densities are 27,275 and 13,102 cones/mm^2^ in the normal eye and the eye with RP, respectively. Proportions of 6-sided Voronoi polygons were 54.1% and 40.0% in the normal eye and the eye with RP, respectively. The ratio of observed average nearest-neighbor distance (NND) for each subject divided by expected NND was 0.795 and 0.723 in the normal eye and the eye with RP, respectively.

To assess the spatial organization of the cone mosaics, the nearest-neighbor distances (NNDs) and Voronoi domains associated with the cones in each mosaic were examined (Fig. 2). Voronoi domains were constructed for each cell by defining points in the regions that were closer to that cell than to any other cell in the mosaic. The ratio of hexagonal Voronoi domains is supposed to express the regularity of cellular arrangement. The NNDs were determined by calculating the minimum distances from the center of that cell to the centers of every other cell in the mosaic. Expected NND was calculated as the expected value for a perfectly hexagonally packed mosaic with a density equal to that in each location [38].

Cone density, ratio of hexagonal Voronoi domain, and average NND/expected NND were determined as the mean value of two independent graders. If the values were significantly different between the graders, a third grader was invited and the value closest to that determined by the third grader was selected. Images with poor image quality were excluded from analysis. If both eyes were eligible, one eye was selected randomly for analysis.

### Spectral-Domain Optical Coherence Tomography: Photoreceptor Layer Features and Retinal Thickness Measurements

**Figure 3:**
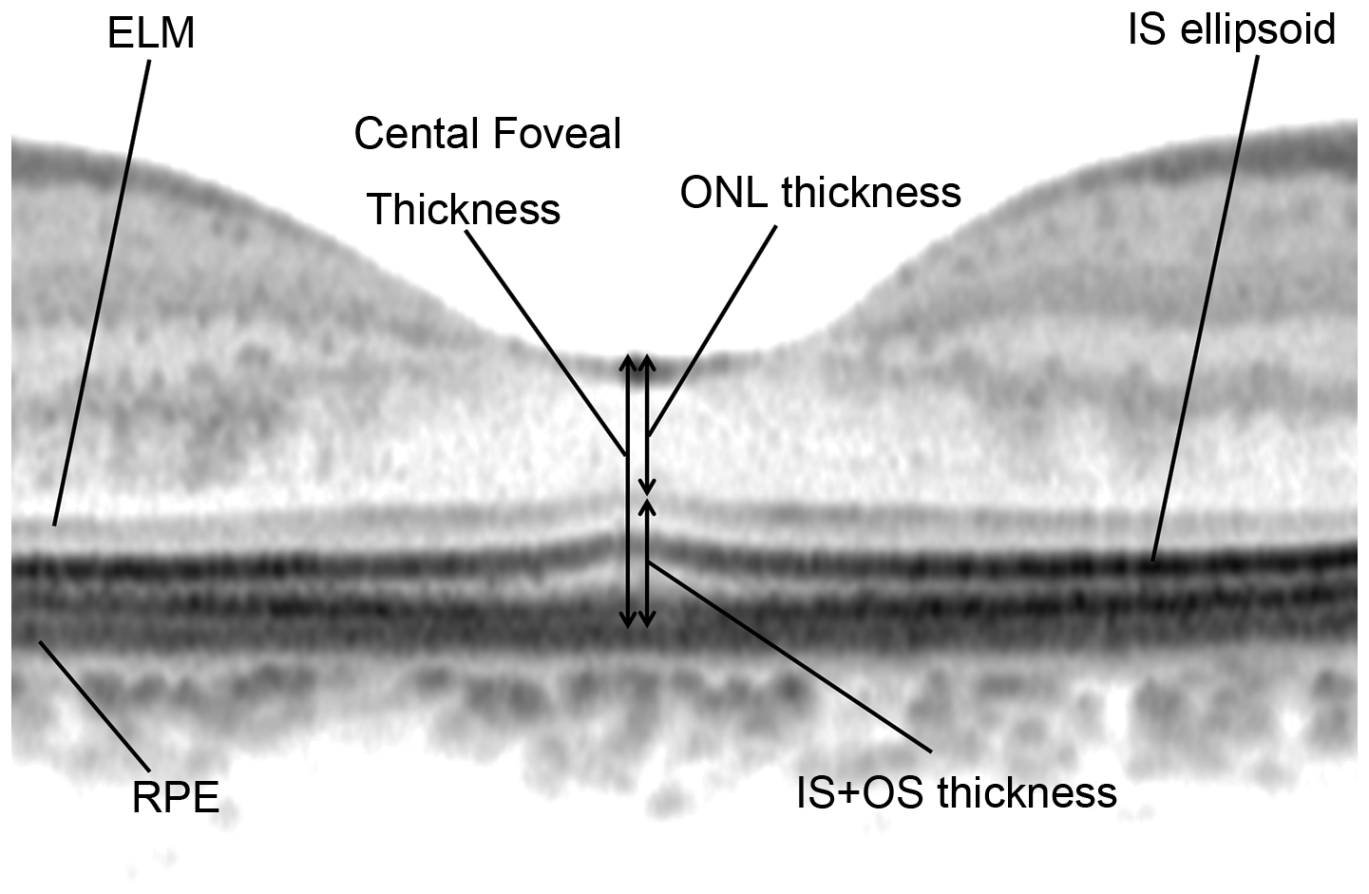
Spectral-domain optical coherence tomography measurement. Measurements included the thickness of the outer nuclear layer (ONL), which was measured between the vitreoretinal interface and external limiting membrane (ELM), the photoreceptor inner segment and outer segment (IS+OS) thickness, measured between the ELM and retinal pigment epithelium (RPE), and the total central foveal thickness.

We used the Spectralis HRA+OCT system (Heidelberg Engineering, Dossenheim, Germany) to perform SD-OCT in all patient eyes. We obtained and evaluated horizontal and vertical B-scan images (30 degrees) through the fovea of each eye. At each location of interest on the retina, 100 SD-OCT images were acquired and averaged to reduce speckle noise. The Spectralis HRA+OCT has a built-in digital caliper to measure thickness or length. We measured the thickness of the outer nuclear layer (ONL; the distance between the vitreoretinal interface and external limiting membrane [ELM]), thickness of the inner segment (IS)+outer segment (OS) (the distance between the ELM and the inner border of the RPE), and the total foveal thickness (the distance between the vitreoretinal interface and the inner border of the RPE) at the center of the fovea (Fig. 3). ONL, IS+OS, and total foveal thickness were determined as the mean value of the measurements made by 2 independent graders. To assess the reproducibility of these measurements, the values obtained by both graders were compared. Features of the status of IS ellipsoid at 0.5 mm from the center of the fovea in each of 4 directions (superior, lower, nasal, and temporal) on SD-OCT images were evaluated by an observer who was masked to the AO-SLO results.

### Humphrey Field Analyzer (10-2 SITA standard program)

We used the Humphrey Field Analyzer (HFA; Carl Zeiss Meditec, Inc. Dublin, CA) to evaluate macular sensitivity. We used not only mean deviation (MD) but also mean total deviation of the central 4 points for analysis; mean total deviation of these central 4 points reflects central sensitivity at a 2°×2° area centered at the fovea [39].

### Statistical Analysis

Best-corrected visual acuity measured using the Landolt chart was expressed as the logarithm angle of resolution (logMAR). We compared age, logMAR, axial length, cone density, and NND and Voronoi parameters using the Mann-Whitney *U* test. The inter-observer interclass correlation coefficients (ICCs) were calculated for measuring cone density, ratio of hexagonal Voronoi domain and average NND/expected NND. The inter-observer ICCs were also calculated for measurements of total foveal thickness, ONL, and IS+OS obtained by SD-OCT. We calculated the Spearman rank correlation coefficient to determine associations between cone density and logMAR, remaining IS ellipsoid size, retinal thickness, retinal sensitivity, and NND and Voronoi parameters. Statistical analyses were performed using SPSS software version 19.0 (SPSS, Inc., Chicago, IL). *P* values less than 0.05 were considered statistically significant.

## Results

The groups of patients and volunteers in this study did not statistically differ in age (42.6±12.5 y for patients; 38.2±6.8 y for volunteers; *P* = 0.211, Mann-Whitney *U* test), logMAR BCVA (−0.11±0.07 for patients; −0.17±0.06 for volunteers; *P* = 0.538, Mann-Whitney *U* test), or axial length (24.3±1.3 mm for patients; 25.1±0.7 mm for volunteers; *P* = 0.213, Mann-Whitney *U* test). Reduced flicker response was observed on full-field ERG or focal macular ERG in all patients (Table 1). However, the amplitude did not decrease below the threshold level (1 µV for flicker ERG according to ISCEV protocol and 0.05 µV for focal macula ERG) in any eyes. Six eyes had mean deviation (MD)<−10 dB, and 8 eyes had MD≥−10 dB. However, mean total deviation (TD) of central 4 points was −2.75 dB (range, −13.25–0 dB) (Table 1). This study included no instance of unreliable HFA results (fixation-loss scores ≥20% or false-positive or false-negative errors ≥33%).

**Table 1:** Clinical characteristics of patients with retinitis pigmentosa.

Case	Age(y)	Sex	Inheritance pattern	Eye	Visual acuity	Axial length (mm)	ERG (cone flicker)		HFA10-2		Cone density0.5 mm from the central fovea (cells/mm^2^)	Cone density1.0 mm from the central fovea (cells/mm^2^)
							Amplitude(µV)	Latency(ms)	MD (dB)	Average of central 4 points of total deviation (dB)		
1	46	M	sporadic	L	20/15	23.09	68.6	28.0	−0.90	−1.00	17,151	13,488
2	47	F	sporadic	L	20/15	24.90	93.6	31.6	−28.30	−4.75	15,583	15,775
3	27	F	AR	L	20/20	25.05	34.0	29.4	−16.37	−13.00	14,707	10,325
4	55	F	sporadic	R	20/15	25.30	25.0	36.6	0.75	−1.75	16,862	12,732
5	43	F	AD	R	20/20	25.80	8.0	30.2	−2.48	−0.50	11,996	10,916
6	51	F	unknown	L	20/15	23.57	31.8	41.8	−11.20	−1.50	17,337	13,799
7	57	F	sporadic	R	20/15	22.24	72.0	30.2	−13.46	−1.00	18,290	15,845
8	33	M	sporadic	L	20/15	23.60	9.1	47.2	−5.77	−1.00	19,433	12,691
9	39	M	AR	R	20/15	25.87	9.3	36.8	−33.69	−13.25	11,202	13,656
10	16	M	AD	R	20/15	22.50	12.6	45.0	−4.21	0	19,321	16,001
11	46	F	AR	R	20/15	23.07	29.3	34.6	0.34	0.75	24,934	18,272
12	63	F	sporadic	L	20/15	24.05	44.75	29.8	−22.83	0	14,697	15,673
13	39	M	AD	L	20/15	25.47	1.5*	49.9*	−4.01	0	12,877	7,843
14	35	F	sporadic	R	20/15	25.86	15.0	29.2	−3.07	−1.5	12,415	10,253

ERG: electroretinogram, HFA: Humphrey Field Analyzer, MD: mean deviation, AR: autosomal recessive, AD: autosomal dominant.

* :result from focal ERG.

In all healthy volunteers, there was no abnormal FAF. In eyes with RP, FAF revealed hyperautofluorescence rings in the macula area in 8 eyes, hypoautofluorescent rings outside hyperautofluorescence in the macula area in 2 eyes, swirls of hyper-autofluorescent in the macula area in 1 eye, and no remarkable abnormalities in the macula area in 3 eyes.

**Figure 4:**
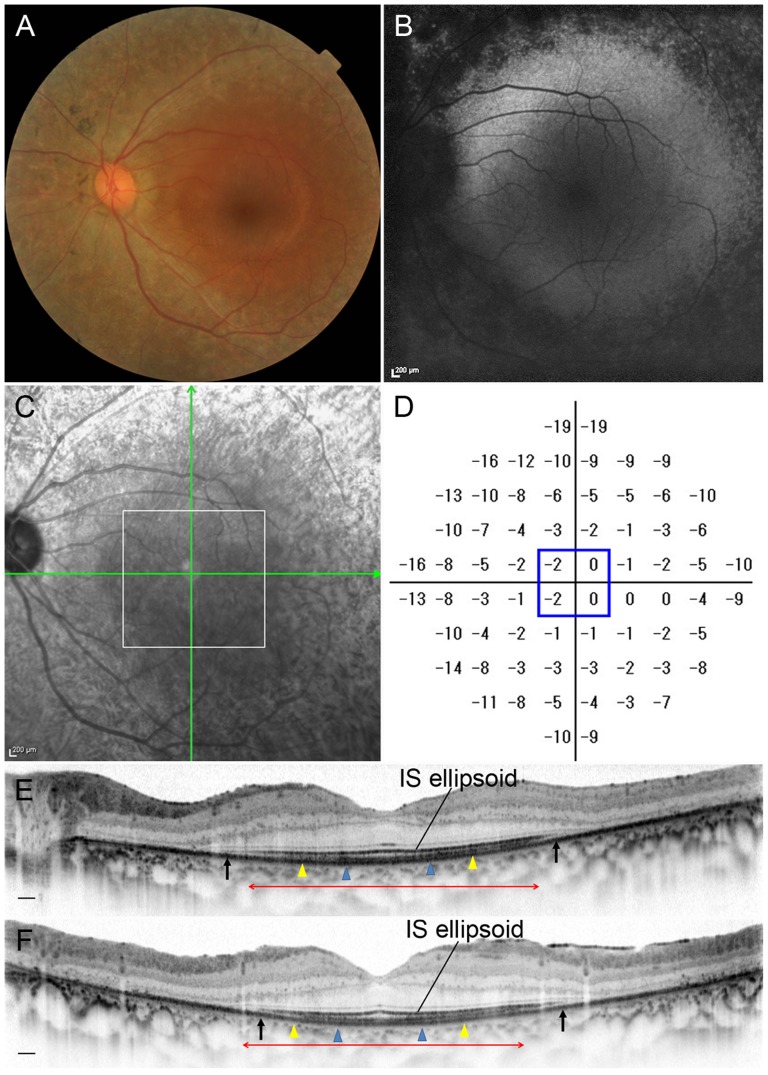
Retinitis Pigmentosa Case (Case 8). Images of the left eye of a 33-year-old man with RP (Case 8). Snellen equivalent best-corrected visual acuity (BCVA) was 20/15. (A) Fundus photograph shows attenuation of retinal vessels and mottling and granularity of the retinal pigment epithelium. (B) FAF image shows hypofluorescenct lesions outside the macula, but normal within the macula. (C) Infrared image with green arrows indicating the directions of scans shown in D and E, and a white box indicating the area scanned by AO-SLO. (D) Total deviation of Humphrey Field Analyzer (10-2 SITA standard program). Blue box indicates the central 4 points. (E) Horizontal SD-OCT line scan through the fovea. (F) Vertical SD-OCT line scan through the fovea. Blue arrowheads indicate 0.5 mm from the center of the fovea, and yellow arrowheads indicate 1.0 mm area from the center of the fovea. IS ellipsoid is remaining in the area between arrows. Red double-headed arrows indicate the area corresponding to the area scanned by AO-SLO.

**Figure 5:**
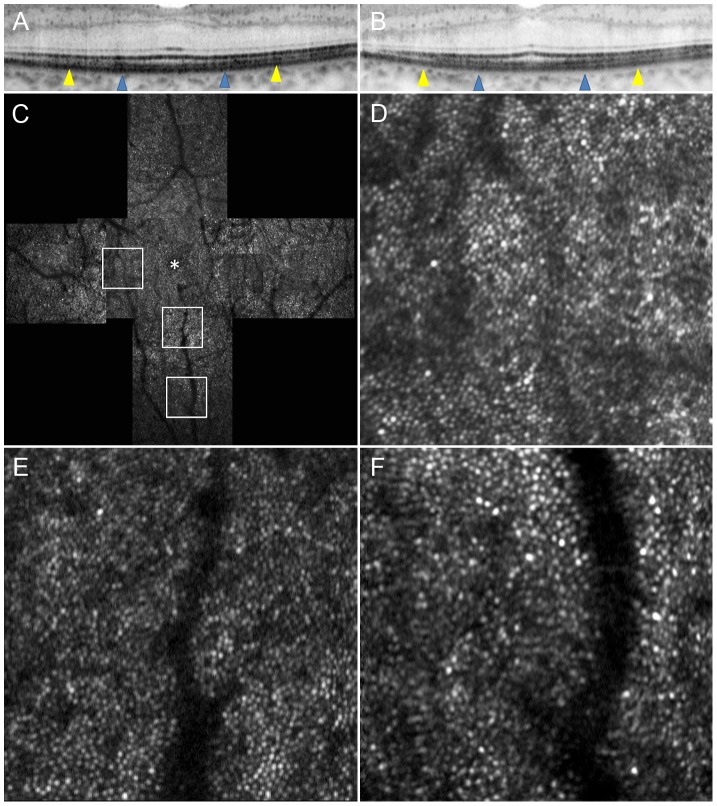
Adaptive Optics Scanning Laser Ophthalmoscopy Image of Case 8. (A) OCT image in a high magnification view of horizontal scan corresponding to the area scanned by AO-SLO. (B) OCT image in a high magnification view of vertical scan corresponding to the area scanned by AO-SLO. Blue arrowheads indicate 0.5 mm from the center of the fovea, and yellow arrowheads indicate 1.0 mm area from the center of the fovea. (C) AO-SLO montage image. The images show cones with a mostly regular mosaic pattern with small dark areas. Small dark areas are seen even in the area where the IS ellipsoid is continuous on SD-OCT (Fig. 4). (D) A high-magnification image at 0.5 mm in the nasal direction from the center of the fovea. (E) A high-magnification image at 0.5 mm in the inferior direction from the center of the fovea. (F) A high-magnification image at 1.0 mm in the inferior direction from the center of the fovea. The asterisk indicates the foveal center.

**Figure 6:**
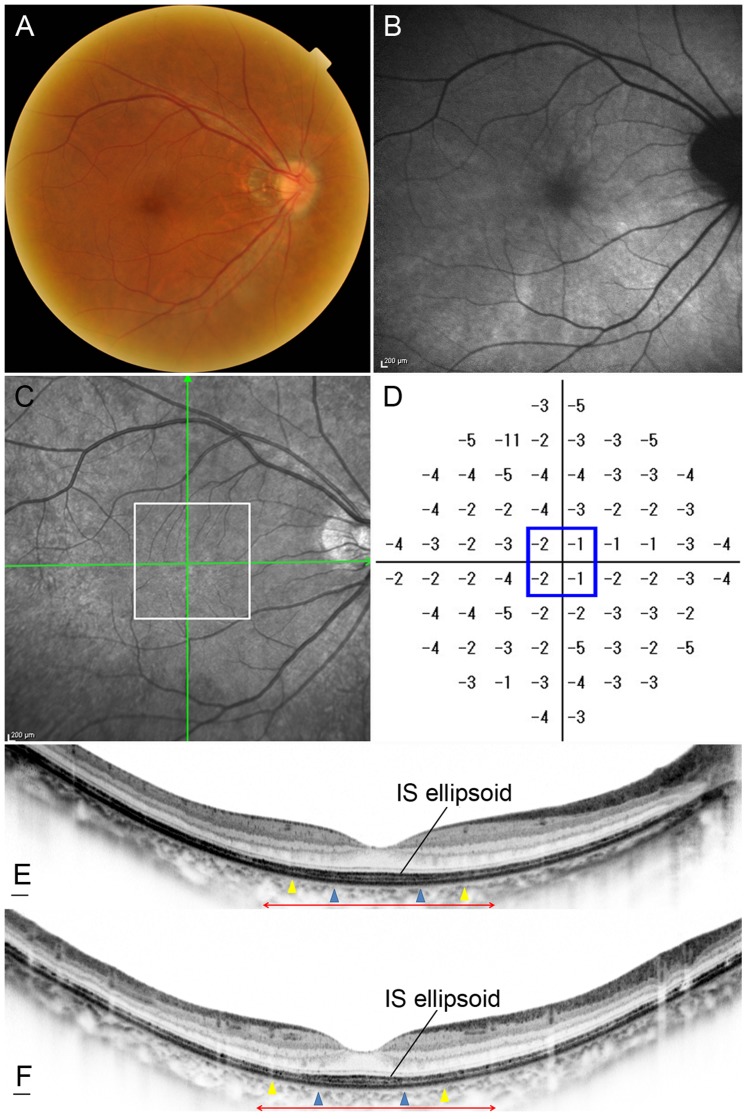
Retinitis Pigmentosa Case (Case 14). Images of the right eye of a 35-year-old female with RP (Case 14). Snellen equivalent BCVA was 20/15. (A) Fundus photograph shows attenuation of retinal vessels and mottling and granularity of the retinal pigment epithelium. (B) FAF image shows swirls of hyperautofluorescence in the macula. (C) Infrared image with green arrows indicating the directions of scans shown in E and F, and a white box indicating the area scanned by AO-SLO. (D) Total deviation of Humphrey Field Analyzer (10-2 SITA standard program). Blue box indicates the central 4 points. (E) Horizontal SD-OCT line scan through the fovea. (F) Vertical SD-OCT line scan through the fovea. Blue arrowheads indicate 0.5 mm from the center of the fovea, and yellow arrowheads indicate 1.0 mm from the center of the fovea. Note that the IS ellipsoid is almost continuous in each scan. Red double-headed arrows indicate the area corresponding to the area scanned by AO-SLO.

**Figure 7:**
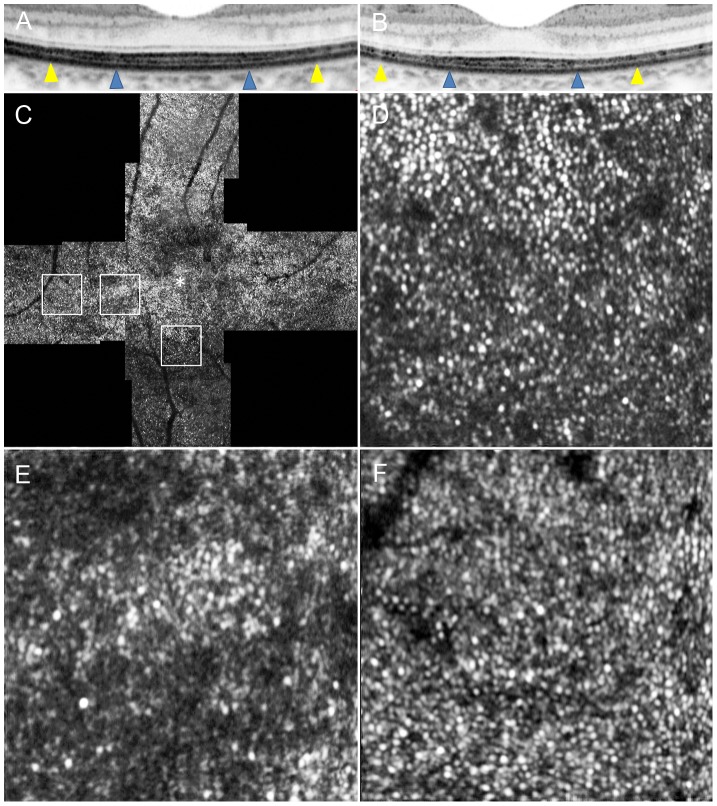
Adaptive Optics Scanning Laser Ophthalmoscopy Image of Case 14. Images of Case 14. (A) OCT image in a high magnification view of horizontal scan corresponding to the area scanned by AO-SLO. (B) OCT image in a high magnification view of vertical scan corresponding to the area scanned by AO-SLO. Blue arrowheads indicate 0.5 mm from the center of the fovea, and yellow arrowheads indicate 1.0 mm area from the center of the fovea. (C) AO-SLO images of Case 14. The images show cones with patchy dark areas representing cone loss. Dark areas are seen even in the area where the IS ellipsoid is continuous on SD-OCT (Fig. 6). (D) A high-magnification image at 0.5 mm in the inferior direction from the center of the fovea. (E) A high-magnification image at 0.5 mm in the temporal direction from the center of the fovea. (D) A high-magnification image at 1.0 mm in the temporal direction from the center of the fovea. The asterisk indicates the foveal center.

**Figure 8:**
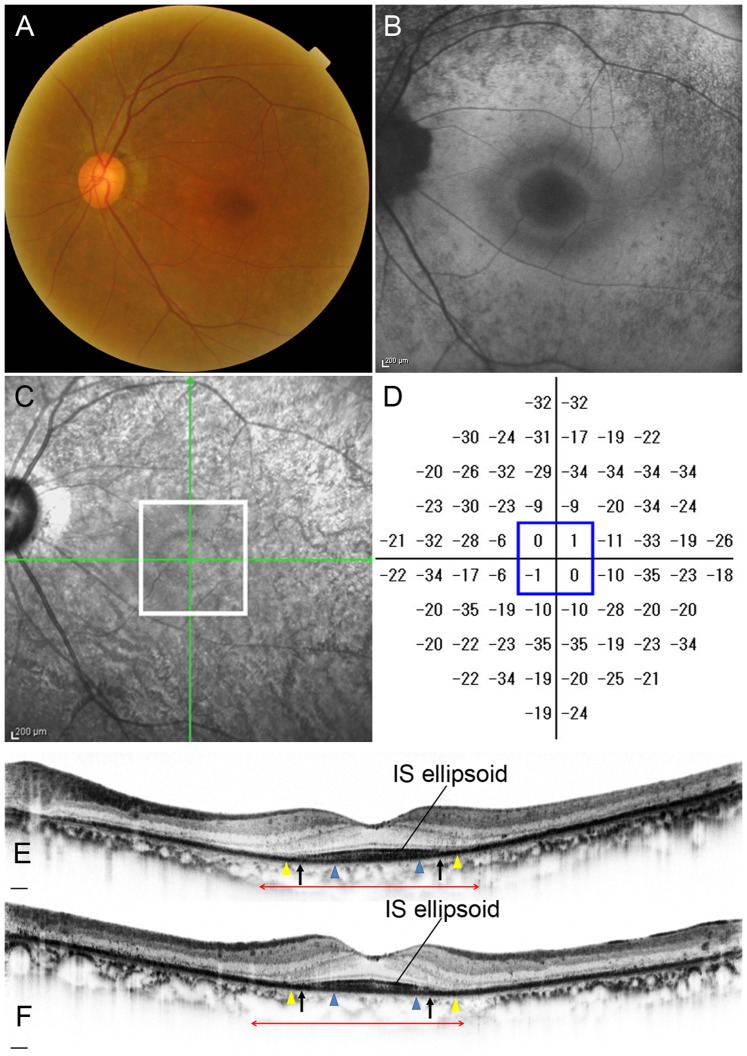
Retinitis Pigmentosa Case (Case 12). Images of the left eye of a 63-year-old female with RP (Case 12). Snellen equivalent BCVA was 20/15. (A) Fundus photograph shows attenuation of retinal vessels and mottling and granularity of the retinal pigment epithelium. (B) FAF image shows a hyperautofluorescent ring surrounded by a hypoautofluorescent ring in the macula. (C) Infrared image with green arrows indicating the directions of scans shown in E and F, and a white box indicating the area scanned by AO-SLO. (D) Total deviation of Humphrey Field Analyzer (10-2 SITA standard program). Blue box indicates the central 4 points. (E) Horizontal SD-OCT line scan through the fovea. (F) Vertical SD-OCT line scan through the fovea. Blue arrowheads indicate 0.5 mm from the center of the fovea, and yellow arrowheads indicate 1.0 mm from the center of the fovea. The IS ellipsoid is remaining in the area between arrows. Red double-headed arrows indicate the area corresponding to the area scanned by AO-SLO.

**Figure 9:**
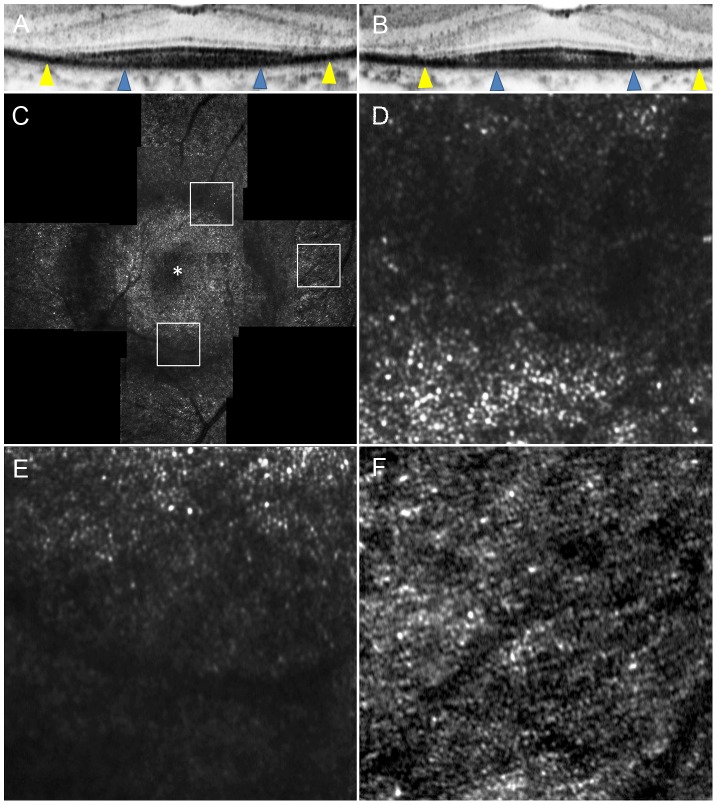
Adaptive Optics Scanning Laser Ophthalmoscopy Image of Case 12. Images of Case 12. (A) OCT image in a high magnification view of horizontal scan corresponding to the area scanned by AO-SLO. (B) OCT image in a high magnification view of vertical scan corresponding to the area scanned by AO-SLO. Blue arrowheads indicate 0.5 mm from the center of the fovea, and yellow arrowheads indicate 1.0 mm area from the center of the fovea. (C) AO-SLO montage images of Case 12. The images show a large dark annular lesion (arrows) where cones are missing, which corresponds to the area where IS ellipsoid is disrupted on SD-OCT (Fig. 8). (D) A high-magnification image at 0.5 mm in the superior direction from the center of the fovea. (E) A high-magnification image at 0.5 mm in the inferior direction from the center of the fovea. (F) A high-magnification image at 1.0 mm in the temporal direction from the center of the fovea. The asterisk indicates the foveal center.

In 3 eyes (21.5%) with RP and preserved central vision, cones formed a mostly regular mosaic pattern with small patchy dark areas (Figs. 4 and 5). In 10 eyes (71.5%), the cone mosaic patterns were less regular, and large dark regions with missing cones were apparent (Figs. 6–9). Only one eye (7.0%) demonstrated a normal, regular cone mosaic pattern.

The inter-observer ICCs were 0.955 for measurements of cone density, 0.811 for the hexagonal Voronoi domain, and 0.951 for the ratio of average-to-expected NND in normal eyes (Table 2). The inter-observer ICCs were 0.980 for measurements of cone density, 0.697 for the hexagonal Voronoi domain, and 0.923 for the ratio of average-to-expected NND in eyes with RP (Table 2).

**Table 2:** The inter-later reliability of intraclass correlation coeffients for the two observers on AO-SLO measurements.

		Intraclass correlation coeffients	
		The inter-later reliability	95% confidence interval
**Normal**	Cone density (cones/mm^2^)	0.955	0.931–0.971
	Ratio of hexagonal Voronoi domain(%)	0.811	0.712–0.876
	Average NND/expected NND	0.951	0.909–0.971
**RP**	Cone density(cones/mm^2^)	0.980	0.966–0.988
	Ratio of hexagonal Voronoi domain(%)	0.697	0.520–0.809
	Average NND/expected NND	0.923	0.834–0.959

NND: nearest-neighbor distance, RP: retinitis pigmentosa.

As shown in Tables 3 and 4, cone density decreased with increasing distance from the center of the fovea in both normal eyes and eyes with RP and preserved central vision; however, cone density was significantly lower in eyes with RP in each area at 0.5 mm and 1.0 mm from the center of the fovea compared to normal eyes (all *P* values<0.05, Mann-Whitney *U* test) except for temporal side 1.0 mm from the center of the fovea.

**Table 3:** Cone density and arrangement in eyes with retinitis pigmentosa vs. normal eyes.

		Eyes with RP (14 eyes of 14 patients)	Normal eyes (12 eyes of 12 volunteers)	*P* *
Age (y)		42.64±12.46	38.17±6.84	0.211
logMAR VA		−0.11±0.07	−0.17±0.06	0.538
**0.5 mm**	Cone density (cones/mm^2^)	16,200±3,683	22,237±1,900	**<0.001**
	Ratio of hexagonal Voronoi domain (%)	39.9±3.1	44.2±2.9	**0.002**
	Average NND/expected NND	0.67±0.03	0.72±0.02	**<0.001**
**1.0 mm**	Cone density (cones/mm^2^)	13,376±2,840	15,975±1,040	**0.021**
	Ratio of hexagonal Voronoi domain (%)	40.4±3.4	46.8±4.9	**0.002**
	Average NND/expected NND	0.66±0.04	0.73±0.02	**<0.001**

* Mann-Whitney *U* test.

VA: visual acuity, NND: nearest-neighbor distance, RP: retinitis pigmentosa.

**Table 4:** Cone density in each region in eyes with retinitis pigmentosa vs. normal eyes.

Distance from central fovea/hemisphere	Eyes with RP (14 eyes of 14 patients) (cones/mm^2^)	Normal eyes (12 eyes of 12 volunteers) (cones/mm^2^)	*P* *
**0.5 mm**			
Superior	15,115±3,857	22,195±4,056	**<0.001**
Nasal	18,123±4,413	22,870±2,741	**0.006**
Inferior	15,221±4,828	21,921±1,680	**0.002**
Temporal	16,868±4,555	21,963±2,950	**0.003**
**1.0 mm**			
Superior	13,319±2,658	15,526±1,245	**0.023**
Nasal	13,466±3,573	16,742±1,370	**0.007**
Inferior	11,994±2,948	14,229±1,405	**0.024**
Temporal	14,629±3,444	17,428±3,059	0.099

* Mann-Whitney *U* test.

RP: retinitis pigmentosa.

Figure 2 and Table 3 show the results of the Voronoi and NND analyses for normal eyes and eyes with RP. At 0.5 mm from the center of the fovea, 44.2% of cones in normal mosaics had 6 neighbors (indicating a regularly packed mosaic), while 39.9% of cones in eyes with RP had 6 neighbors (*P* = 0.002, Mann-Whitney *U* test). At 1.0 mm from the center of the fovea, 46.8% of cones in normal mosaics and 40.4% of cones in eyes with RP had 6 neighbors (*P* = 0.002, Mann-Whitney *U* test). The ratio of observed average NND for each subject divided by expected NND (computed assuming a perfectly hexagonal lattice of cones with a density equal to that observed for a given subject) was significantly lower for eyes with RP than for normal eyes at 0.5 mm and 1.0 mm from the center of the fovea (*P*<0.001 for both, Mann-Whitney *U* test).

Comparison of AO-SLO and SD-OCT images in eyes with RP showed that large dark regions in the AO-SLO images corresponded to areas in the SD-OCT images where the line representing the IS ellipsoid was disrupted (Figs. 8 and 9). In eyes with RP, greater decrease in cone density 0.5 mm from the center of the fovea was related to disruption of the line representing the IS ellipsoid on SD-OCT images (*P* = 0.044, Mann-Whitney *U* test; Table 5). However, small patchy dark regions were seen on AO-SLO even in areas with continuous IS ellipsoid on SD-OCT (Figs. 4–7).

**Table 5:** Cone density and arrangement on AO-SLO vs. photoreceptor status on SD-OCT in eyes with retinitis pigmentosa (0.5 mm from center of the fovea).

	Intact IS ellipsoid	Disrupted IS ellipsoid	*P* *
Cone density (cones/mm^2^)	16,784±4,567	13,170±2,715	**0.044**
Ratio of hexagonal Voronoi domain (%)	40.1±6.2	38.4±4.8	0.479
Average NND/expected NND	0.68±0.04	0.66±0.04	0.276

* Mann-Whitney *U* test.

IS: photoreceptor inner segment, NND: nearest-neighbor distance.

For SD-OCT measurements, the inter-observer ICCs were 0.999, 0.996, and 0.980 for measurements of total foveal thickness, ONL, and IS+OS, respectively (Table 6).

**Table 6:** The inter-later reliability of intraclass correlation coeffients for the two observers on SD-OCT measurements.

	Intraclass correlation coeffients	
	The inter-later reliability	95% confidence interval
Total foveal thickness	0.999	0.996–1.000
ONL	0.996	0.591–0.999
IS+OS	0.980	0.919–0.994

ONL: outer nuclear layer, IS: photoreceptor inner segment, OS: photoreceptor outer segment.

In eyes with RP with preserved central vision, cone density did not correlate with HFA results (Table 7). However, decreased cone density correlated thinner ONL (*P* = 0.029) and thinner IS+OS (*P* = 0.011), and thinner total foveal thickness (*P* = 0.008) on SD-OCT (Table 8).

**Table 7:** Correlation between the result of AO-SLO or SD-OCT and retinal sensitivity in eyes with retinitis pigmentosa (0.5 mm from center of the fovea).

		HFA 10-2	
		MD	Mean TD of central 4 points
**AO-SLO**	Cone density (cones/mm^2^)	*P* = 0.375 *rs* = 0.26	*P* = 0.201 *rs* = 0.36
	Ratio of hexagonal Voronoi domain(%)	*P* = 0.771 *rs* = 0.09	*P* = 0.982 *rs* = 0.01
	Average NND/expected NND	*P* = 0.309 *rs* = 0.29	*P* = 0.304 *rs* = 0.30
**SD-OCT**	ONL	*P* = 0.513 *rs* = 0.19	*P* = 0.427 *rs* = 0.23
	IS+OS Thickness	*P* = 0.140 *rs* = 0.42	*P* = 0.052 *rs* = 0.53

Spearman's correlation.

VA: visual acuity, HFA: Humphrey Field Analyzer, MD: mean deviation,

TD: total deviation, ONL: outer nuclear layer, IS: photoreceptor inner segment,

OS: photoreceptor outer segment.

**Table 8:** Correlation between cone density/arrangement and OCT findings in eyes with retinitis pigmentosa.

	Cone density (cones/mm^2^)	Ratio of hexagonal Voronoi domain (%)	Average NND/expected NND
Total foveal thickness (µm)	***P*** ** = 0.008 ** ***rs*** ** = 0.679**	*P* = 0.267 *rs* = 0.32	*P* = 0.061 *rs* = 0.51
ONL thickness (µm)	***P*** ** = 0.029 ** ***rs*** ** = 0.58**	*P* = 0.122 *rs* = 0.43	*P* = 0.227 *rs* = 0.35
IS+OS thickness (µm)	***P*** ** = 0.011 ** ***rs*** ** = 0.66**	*P* = 0.114 *rs* = 0.44	***P*** ** = 0.029 ** ***rs*** ** = 0.58**
Width of remaining IS ellipsoid	*P* = 0.483 *rs* = 0.20	*P* = 0.911 *rs* = 0.03	*P* = 0.349 *rs* = 0.27

Spearman's correlation.

NND: nearest-neighbor distance, ONL: outer nuclear layer, IS: inner segment of photoreceptor, OS: outer segment of photoreceptor.

## Discussion

Retinal degeneration in RP is characterized by slowly progressive death of rod and cone photoreceptors. Studies using OCT images obtained in eyes with RP have revealed structural abnormalities in these eyes, including disruption of the line representing the IS ellipsoid, intraretinal cystic spaces, and thinning of the OPL/ONL and the IS+OS thickness [6]–[8], [10], [40]–[43]. However, these studies have not ascertained how individual cones are damaged in RP.

Several researchers have reported the observation of abnormal cone patterns using AO-imaging devices in eyes with forms of inherited retinal degeneration, including cone-rod dystrophy, T8993C mitochondrial DNA mutation, Stargardt disease, and RP [17]–[21], [44]. These eyes demonstrated large areas devoid of cones within atrophic regions and patchy cone mosaics due to photoreceptor dropout. Talcott et al included 4 eyes with RP with BCVA of 20/20 or better [21]. Unfortunately, the report did not include the individual AO imaging data for each patient. Choi et al included 2 eyes with rod-cone dystrophy with BCVA of 20/20 or better [18]. Cone density was 74% and 59% at 2° temporal from the fovea. Duncan et al. included 5 eyes from 5 patients with RP, 4 of which had BCVA of 20/20 or better [19]. At 1° from the fovea, the mean cone spacing was 20% greater among the RP patients as compared to normal subjects; this difference was even greater in several eyes with BCVA of 20/20 or better. These results are quite similar to our findings, although these studies included limited sample sizes. Our cross-sectional case series, which included and focused on a larger number of patients with RP with BCVA of 20/20 or better, have demonstrated the clinical relevancy of these findings.

Voronoi and nearest-neighbor analyses are widely used to assess the regularity of cellular mosaics in the retina [36], [45]. In the current study, a smaller number of cones in eyes with RP had 6 neighbors compared to cones in normal eyes, indicating an irregularly shaped mosaic. In addition, the ratio of observed mean NND to expected NND was significantly lower for eyes with RP than for normal eyes, suggesting a marked departure from perfect arrangement of the cone mosaic. Thus, the regularity of the spatial arrangement of the cone mosaic is disrupted in eyes with RP, even when visual acuity and foveal sensitivity are good.

In the current study, we further compared AO-SLO findings with SD-OCT findings in eyes with RP. We found a correlation between SD-OCT evidence of a disrupted IS ellipsoid and AO-SLO findings of disruption of the cone mosaic pattern, as indicated by dark regions. In eyes with RP, greater decrease in cone density was related to a larger area of disruption in the line representing the IS ellipsoid in SD-OCT images. These results are consistent with the results of previous studies of eyes with macular microholes, resolved central serous chorioretinopathy, or idiopathic macular telangiectasia, in which the dark area seen in the AO images corresponded with the areas where the line representing the IS ellipsoid or the cone outer segment tip was disrupted in corresponding SD-OCT images [25], [28]. In contrast, Voronoi and nearest-neighbor analyses did not differ regardless of IS ellipsoid status. Thus, in eyes with RP, IS ellipsoid irregularities or disruptions on SD-OCT do not suggest cone disarrangement but rather cone loss.

The small patchy dark regions were also seen on AO-SLO, even in areas with continuous IS ellipsoid as revealed by SD-OCT. We believe our inability to detect the small patchy dark regions seen in AO-SLO using SD-OCT results from resolution differences. The small dark regions we saw using AO-SLO, which has a lateral resolution of ∼5 µm, were approximately 5–20 µm across, whereas the lateral resolution of commercially available SD-OCT systems, which do not have AO, is approximately 20 µm. On the other hand, SD-OCT is more sensitive than AO-SLO. In the current study, photoreceptor layers that could be visualized using the SD-OCT were not reflective enough to be seen in some AO-SLO images. Further study is needed to compare AO-SLO montage images and IS ellipsoid maps obtained by SD-OCT.

Lower cone density on AO-SLO correlated thinner ONL and photoreceptor layer on SD-OCT in eyes with RP. Thus, decreased ONL and photoreceptor thickness may reflect more severe structural disturbance of the photoreceptor layer than the IS ellipsoid status. In fact, using SD-OCT, several researchers have shown that ONL and photoreceptor thickness decreased with loss of local field sensitivity in RP [8], [46]–[49]. Wen et al. reported that preserved cone function measured by multifocal ERG amplitude and visual field sensitivity correlated with the remaining thickness of the photoreceptor layer in patients with RP [10].

Natural history studies of retinal degeneration predict that significant changes in visual function may be measured reliably only after more than 7 years [3]. The lack of sensitive outcome measures of disease progression may have hampered the development of treatments for RP. However, Talcott et al. recently reported that direct observation and analysis of cone structure on AO-SLO images may provide a sensitive measure of disease progression and treatment response in patients with inherited retinal degeneration [21]. Our results also suggest that AO-SLO is a useful tool to detect macular cone abnormalities with greater sensitivity than standard measures of visual function in eyes with preserved central vision. Thus, further studies of cone structure using AO-SLO are needed to evaluate the effect of experimental treatments such as neurotrophic factors, especially during the stage of preserved central vision.

Our study has several limitations. (1) Although our AO imaging equipment has better lateral resolution than commercially available SD-OCT, it was still unable to clearly show individual cone photoreceptors in the foveal center, which made it difficult to identify a correlation between cone density and visual acuity. Recently, Dubra et al visualized rods and foveal cones using an improved AO-SLO system [50]. These technological improvements may reveal anatomical changes that underlie RP pathology, such as the relationship between foveal cone damage and visual acuity. (2) Most of the healthy subjects were men. However, cone density has not been reported to differ between sexes, though it may vary with distance from the fovea, age, axial length, and reflective error [45], [51], [52]. (3) Gene mutation associated with RP was confirmed in none of the patients included in the study. Despite these limitations, our study shows that AO-SLO imaging is a sensitive and quantitative tool for detecting photoreceptor abnormalities in eyes with RP and preserved central vision. We are therefore planning prospective longitudinal studies using AO-SLO to learn more about cone abnormalities in the progression of RP, with the hope that this knowledge will point the way to better management of this disease.

## Supporting Information

Information S1 **Adaptive Optics Scanning Laser Ophthalmoscopy System.**
(DOCX)
