# Supplementary material for: Macular Cone Abnormalities in Retinitis Pigmentosa with Preserved Central Vision Using Adaptive Optics Scanning Laser Ophthalmoscopy
Source: PLoS One. 2013 Nov 19;8(11):e79447. doi: 10.1371/journal.pone.0079447 (PMC3834127; doi:10.1371/journal.pone.0079447)
Supplement: Information S1 — Adaptive Optics Scanning Laser Ophthalmoscopy System. (DOCX) [file pone.0079447.s001.docx]

**Supporting Information 1: Adaptive Optics Scanning Laser Ophthalmoscopy System**

The AO-SLO system enabling acquisition of high-resolution retinal images consists of the following subsystems for (1) measurement of eye aberration, (2) wavefront compensation of imaging light, (3) scanning and detecting the imaging light, and (4) composition of a SLO image. The details of these subsystems are described below.

The subsystem for measurement of eye aberration consists of a beacon light source and a wavefront sensor. The beacon light source is a superluminescent diode (QSDM-760-2, QPhotonics, USA) with wavelength of 760±5 nm. The incident light emitted from the beacon light source propagates and enters the pupil of an eye with beam width of 1 mm at 40 μW. The beacon light reflected on the retina propagates along the optics via optical elements such as mirrors and then enters the Hartmann-Shack wavefront sensor (HASO32-eye, ImagineEyes, France). The aberration of the eye is calculated with the wavefront data measured by this wavefront sensor.

The subsystem for wavefront compensation of imaging light consists of 2 LCOS-SLMs (X10468-02, Hamamatsu Photonics, Japan). These LCOS-SLMs modulate the phase of the imaging light according to the eye aberration measured by the beacon light and wavefront sensor, and compensate the eye aberration at a high order (Zernike polynomial sixth order) so that the imaging light can focus on the retina. For an eye with large reflective error, a focus lens and a cylindrical lens are arranged on the imaging light path. These lenses function to compensate defocus from -10 to +5 D and astigmatism from 0 to 5 D, respectively.

The subsystem for scanning and detecting the imaging light consists of an imaging light source, a XY optical scanning unit, and a photo detector. The imaging light source is a superluminescent diode (S840-B-I-20, Superlum, Ireland) with wavelength of 840±25 nm. The incident light emitted from the imaging light source propagates along the optics with some mirrors and 2 LCOS-SLMs that compensate the wavefront of the imaging light, and then enters the XY optical scanning unit composed of an 8-kHz resonant scanner (CRS 8 kHz, GSI Group, USA) and a galvano scanner (VM500+, GSI Group, USA). These scanners scan the imaging light on the retina in the vertical and horizontal directions, respectively. The scanning areas on a retina are chosen from 0.34 mm × 0.34 mm (32 frames per second [fps]), 0.82 mm × 0.82 mm (32 fps), 1.7 mm × 1.7 mm (32 fps), 0.34 mm × 0.17 mm (64 fps), 0.82 mm × 0.41 mm (64 fps), and 1.7 mm × 0.85 mm (64 fps). The imaging light scanned by XY scanner enters the pupil of the eye with a beam width of 4 mm at 330 μW. The resolution in Rayleigh criterion, which is calculated from wavelength and beam width, is approximately 5 μm. The power of incident light from the beacon light and imaging light was determined in consideration of ANSI safety limits. The imaging light reflected on the retina propagates almost the same optical path of the incident light beam, and reflects off of a half mirror to enter the photo detector. The photo detector is an avalanche photodiode (C5460, Hamamatsu Photonics, Japan), which converts the detected light power to an electric signal.

The subsystem for composition of a SLO image consists of an amplifier, an AD board, and a personal computer. The electrical signal that is converted from the detected light power is amplified by a commercial amplifier (SR560, Stanford Research Systems, USA). The amplified signal is digitized by an AD board, and then the digitized data, which is used to compose the retinal SLO image, is recorded to a hard disk of the personal computer. The composed SLO image size is typically 400 × 400 pixels (32 fps) and 400 × 200 pixels (64 fps).
